# Insight into determinants of substrate binding and transport in a multidrug efflux protein

**DOI:** 10.1038/srep22833

**Published:** 2016-03-10

**Authors:** Kamela O. Alegre, Stephanie Paul, Paola Labarbuta, Christopher J. Law

**Affiliations:** 1 School of Biological Sciences, Medical Biology Centre, Queen’s University Belfast, Belfast BT9 7BL, United Kingdom

## Abstract

Multidrug resistance arising from the activity of integral membrane transporter proteins presents a global public health threat. In bacteria such as *Escherichia coli*, transporter proteins belonging to the major facilitator superfamily make a considerable contribution to multidrug resistance by catalysing efflux of myriad structurally and chemically different antimicrobial compounds. Despite their clinical relevance, questions pertaining to mechanistic details of how these promiscuous proteins function remain outstanding, and the role(s) played by individual amino acid residues in recognition, binding and subsequent transport of different antimicrobial substrates by multidrug efflux members of the major facilitator superfamily requires illumination. Using *in silico* homology modelling, molecular docking and mutagenesis studies in combination with substrate binding and transport assays, we identified several amino acid residues that play important roles in antimicrobial substrate recognition, binding and transport by *Escherichia coli* MdtM, a representative multidrug efflux protein of the major facilitator superfamily. Furthermore, our studies suggested that ‘aromatic clamps’ formed by tyrosine and phenylalanine residues located within the substrate binding pocket of MdtM may be important for antimicrobial substrate recognition and transport by the protein. Such ‘clamps’ may be a structurally and functionally important feature of all major facilitator multidrug efflux proteins.

The phenomenon of multidrug resistance – a serious global public health threat-arises principally from active efflux of drugs out of the cell cytoplasm by proteins that are integral membrane transporters. The ‘transportome’ of most bacteria contains numerous drug efflux proteins^1^, some of which are driven by energy released by ATP hydrolysis (the primary active transporters) and others by the energy stored in electrochemical gradients (the secondary active transporters).

The majority of bacterial drug efflux proteins belong to the ubiquitous, large and diverse major facilitator superfamily (MFS) of secondary active transporters^2^. Although sequence similarity among the MFS is generally poor, all appear to follow the same structural template, their architecture consisting of either 12- or 14-transmembrane (TM) α-helices that are separated into six- or seven-helix bundle N- and C-terminal domains, respectively, related by a pseudo twofold symmetry and connected by a long cytoplasmic loop^3^. The N- and C-terminal halves of the transporter saddle a central substrate translocation pore, and both the N- and C-tails of the protein are located inside the cell. This structural arrangement implies that MFS proteins function via a single binding site, alternating access mechanism that is accompanied by a rocker switch-like movement of the two halves of the transporter, permitting access to the binding site(s) to swap between cytoplasm and periplasm^4^. The rocker switch mechanism supports at least three major conformational states during a dynamic transport cycle: a cytoplasmic or inward-facing conformation (C_in_), an outward-facing one (C_out_), and a more compact, occluded conformation (C_occ_) in the intermediate state^4^.

Six families within the embrace of the MFS contain multidrug efflux transporters^5^, the most frequently occurring of which are those of the 12 or 14 TM segment drug/H^+^ antiporter families (DHA1 and DHA2, respectively) that use the electrochemical ion gradient to drive drugs out of the cell^6^. In stark contrast to the stringent specificity for single substrates within a diverse pool of substrate types (including ions, sugars, organic phosphates, neurotransmitters, nucleosides, amino acids and peptides) displayed by most members of the MFS, the multidrug transporters of the superfamily are polyspecific and can handle and extrude a remarkably diverse range of substrates^7^. Such substrate promiscuity imparts clinical significance to these proteins in that they can represent a barrier to successful treatment of infection.

Extensive biochemical studies of the prototypical *Escherichia coli* MFS drug/H^+^ antiporter, MdfA, suggested the structural basis of substrate promiscuity lies in the presence of a large, flexible and complex substrate recognition cavity within the protein, which permits different substrates to interact with different regions of the cavity, and to form different interactions with it^7^,^8^,^9^,^10^. Interactions between neutral or zwitterionic substrates and the cavity occur primarily via low-specificity hydrophobic interactions, while electrostatic interactions between substrate and a membrane-embedded, negatively charged amino acid side chain of the protein are seemingly important for the transport of cationic compounds^10^,^11^,^12^,^13^,^14^. The picture is further complicated by evidence that two dissimilar substrates can bind simultaneously to distinct but allosterically linked sites within the transporter^15^. Although the crystal structure of EmrD, an *E. coli* MdfA homologue, captured in occluded conformation supports the notion of a large, hydrophobic, substrate-recognition cavity, the lack of any substrate molecule resolved in the structure precluded any detailed interpretation of a structural basis for substrate polyspecificity in this multidrug efflux representative of the MFS^16^. Recently, however, high-resolution structures of an inward-facing conformation of MdfA in complex with substrate have been reported^17^ and these have provided valuable insight into the substrate-binding mode and transport mechanism of MFS multidrug efflux proteins in general. However, despite this much enhanced understanding, questions pertaining to multidrug recognition and transport by members of the MFS are still outstanding; for example, are the same residues involved in substrate recognition and binding in other conformations of the protein? Moreover, are the same residues involved in recognition of all substrate types the protein is capable of transporting? We have attempted to shed light on these questions by performing a combination of *in silico*, biochemical, and mutagenesis studies on the *E. coli* MFS drug/H^+^ antiporter MdtM.

MdtM is a versatile and close relative of MdfA^18^ that not only renders cells expressing it from multicopy plasmid resistant to a range of antimicrobial compounds^19^,^20^,^21^,^22^ but also functions physiologically as a monovalent metal cation/H^+^ antiporter with a role in pH homeostasis in alkaline environments^23^ and in protection of cells against the cytotoxic effects of bile salts^24^. We generated an homology model of MdtM in occluded conformation for use in molecular docking studies of two known antimicrobial substrates of the transporter, one neutral at physiological pH (chloramphenicol) and the other (tetraphenylphosphonium or TPP^+^) cationic, with the aim of identifying amino acid side chains that function in recognition and binding of the differently charged substrates. The results of the *in silico* studies were used to guide subsequent mutagenesis experiments that tested the effects of individual mutations on the drug efflux phenotype, and the substrate binding and transport characteristics of the protein.

## Results

### Homology model of MdtM in occluded conformation

**Figure 1:**
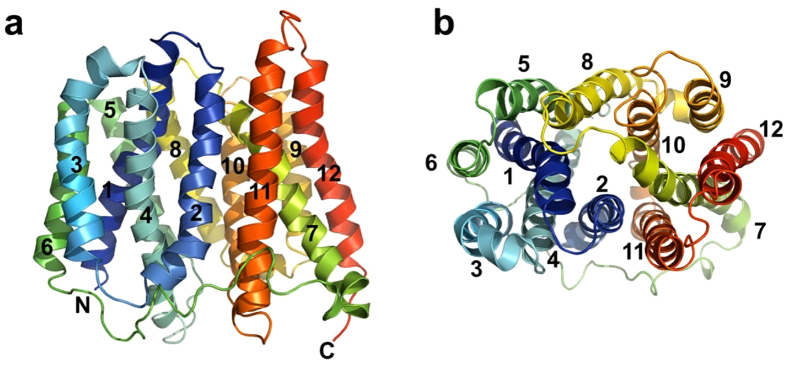
Homology model of MdtM in occluded conformation. (**a**) viewed parallel to the plane of the membrane with the cytoplasmic side at the bottom and the periplasmic side at the top. The N- and C-termini are labelled, and the transmembrane helices are numbered. (**b**) viewed perpendicular to the plane of the membrane from the periplasmic side. Figure was produced using the PyMOL Molecular Graphics System (Schrödinger, LLC).

The homology model of MdtM, which represents the transporter in a compact and apparently occluded conformation that may represent a transition state of the transport cycle^3^, consisted of 379 residues (14–392) corresponding to residues 9–382 of the EmrD template structure (Fig. 1). The sequence identity between EmrD and MdtM over these residues was 20%, compared to the overall sequence identity and similarity between MdtM and EmrD of 23% and 43%, respectively. The N- and C-terminal cytoplasmic tail regions of MdtM (residues 1–13 and 393–410, respectively) were not modelled because the equivalent residues were not resolved in the EmrD crystallographic structure.

The optimised MdtM model was subjected to computational validation analyses to test its reliability and consistency. The stereochemical quality of the model was assessed using PROCHECK validation software, and the resulting Ramachandran plot output revealed that 96.4% of the non-glycine and non-proline amino acid residues of the model were located in the favoured and additionally allowed regions, with only 1.8% each in the generously allowed and disallowed regions (Supplementary Fig. S1). The residues in the latter two regions of the plot are located in periplasmic or cytoplasmic loops of the MdtM model; these loop regions are not expected to be perfectly predicted for membrane proteins. All the main chain parameters of the model are within the typically expected bandwidths and the overall *G-*factor was −0.22. The same analysis performed on the crystallographic structure of the EmrD template yielded similar results, thereby instilling confidence in the overall correctness of the MdtM model.

The overall architecture of the model is typical of MFS transporters^3^ and consists of 12 TM α-helices of generally different lengths arranged into two six-helix, N- and C-terminus bundles that are joined on the cytoplasmic side of the protein by a long loop that extends from TM6 to TM7 (Fig. 1). As shown in Fig. 1b, eight of the TM helices (TM helices 1, 2, 4 and 5 in the N-terminal half of the protein and TM helices 7, 8, 10 and 11 in the C-terminal half) form a palisade around a large, central cavity that represents the substrate-binding region of the transporter. Similar to the crystallographic structures of EmrD^16^ and MdfA^17^, several bulky, aromatic, and polar uncharged residues line the binding cavity of the MdtM model and impart a generally hydrophobic character upon it; at least some of these cavity residues are likely to function in recognition and binding of the drug substrates transported by MdtM.

Comparison of our MdtM model with the inward-open MdfA crystal structure^17^ revealed similarities with respect to the location of individual residues important for transporter function. In MdfA, two membrane embedded, negatively charged residues (E26 and D34) located in TM1, and a positively charged arginine in TM4 (R112) have been implicated in transport function^12^,^17^; indeed, a positive charge at position 112 is essential for MdfA function^25^. As shown in Supplementary Fig. 2, the equivalent residues (D22, D30, and R108) in the MdtM model are also located in TM1 and TM4 and at a very similar depth within the membrane as the corresponding MdfA residues. In MdfA a glutamate (E132) that functions in substrate recognition^25^ caps the cytoplasmic end of TM4 and an equivalent glutamate (E128) is found in the same location in our MdtM model.

### Molecular docking of substrates to the MdtM model

The crystal structures of MdfA revealed amino acid residues involved in substrate binding to the C_in_ conformation of the protein^17^ and, considering the close homology of MdtM to MdfA^21^, it is likely that the equivalent residues in MdtM also function in substrate binding. However, it is unknown if the same residues remain involved as the transporter undergoes conformational change. We therefore used our model of MdtM for molecular docking studies of two representative substrates of the transporter to identify residues potentially involved in binding of substrate to the occluded state.

**Figure 2:**
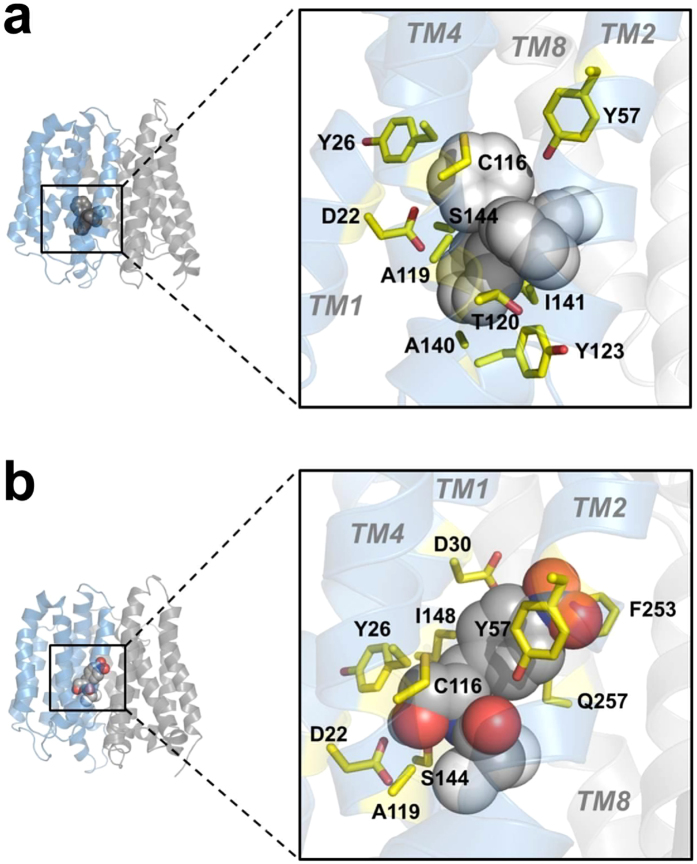
Molecular docking of substrates to the MdtM homology model. (**a**) docking of TPP^+^ to MdtM in occluded conformation. The TPP^+^ substrate is depicted as a sphere model with carbon atoms coloured grey and the interacting protein side chains as yellow sticks. (**b**) docking of chloramphenicol to MdtM in occluded conformation. The substrate is depicted as spheres (with carbon atoms coloured grey, oxygen atoms red, nitrogen atoms blue and chlorine atoms white). Interacting protein side chains are represented as yellow sticks. In (**a**,**b**), the protein is viewed parallel to the membrane plane with the periplasmic side at the top. Transmembrane helices (TMs) that constitute the N-terminal half of the protein are shaded in sky blue and those of the C-terminal half in grey. Figure was produced using the PyMOL Molecular Graphics System (Schrödinger, LLC).

Docking of the TPP^+^ and chloramphenicol substrate molecules enabled visualization of the spatial relationship between the whole MdtM molecule and the substrates (Fig. 2). Both substrates bound in the central cavity of the transporter, but with a clear bias toward the N-terminal domain, in a large pocket formed by the membrane spanning regions of TM1, TM2, TM4 and TM5. Furthermore, although the binding site of each substrate was distinct, some overlap was apparent. These observations are largely consistent with the results of a previous study of substrate binding to MdfA that suggested the existence of distinct but interacting binding sites within a large, flexible substrate recognition pocket of the protein that could accommodate the simultaneous binding of TPP^+^ and chloramphenicol^15^; our docking studies with MdtM suggest this is a common feature of multidrug efflux members of the MFS.

The results of docking TPP^+^ to MdtM revealed that the majority of the molecular interactions between the substrate and the protein were hydrophobic in character, with a complete absence of any H-bonding or polar interactions (Supplementary Table S1). Hydrophobic interactions occurred between TPP^+^ and the MdtM C116, A119, A140 and I141 residues. The aromatic side chains of two tyrosine residues, Y26 and Y123, interacted with two of the four phenyl rings of TPP^+^ via π-π interactions to form an ‘aromatic clamp’, like a pair of tongs, around the substrate molecule (Fig. 2a). The docking study also suggested D22, T120 and S144 of MdtM as interaction partners of the substrate (Supplementary Table S1), with the oxygen atoms of those amino acid side chains perhaps involved in making anion-π interactions with the edge of the TPP^+^ phenyl rings (Fig. 2a).

In contrast to the absence of H-bonding and polar interactions between TTP^+^ and MdtM, these types of interactions were prevalent when chloramphenicol was docked to the transporter (Supplementary Table S2). H-bonding was observed between the substrate and the S144 side chain of MdtM, polar interactions were made with D22, Y57, S144 and Q257, and the A119 side chain also interacted the substrate (Fig. 2b). Additionally, hydrophobic interactions formed between chloramphenicol and the C116 and I148 residues of MdtM. An aromatic triad consisting of Y26, Y57 and F253 interacted with the chloramphenicol molecule via π-π interactions. The aromatic rings of Y57 and F253 appeared to act as a ‘clamp’ to hold the substrate in place in the pore of the transporter. Notably, the interaction between chloramphenicol and Y26, and a further interaction that occurred between substrate and the acidic D30 residue of MdtM in our docking study were consistent with observations from the substrate-bound, inward-open MdfA crystal structure in which the equivalent conserved residues (Y30 and E34) of MdfA were also shown to function in chloramphenicol binding^17^.

The *in silico* studies described above were performed using a static model that represents the MdtM protein structure at one particular phase of the transport cycle. In reality, MdtM is a dynamic protein that undergoes conformational change during the drug efflux event^4^,^26^; it is therefore probable that substrate interacts with other residues as it initially ‘explores’ the binding cavity of the protein^27^,^28^ and subsequently translocates through the pore during the transport event. Such substrate-interacting residues have been identified in other MFS transporters^9^,^11^,^17^,^25^,^29^,^30^,^31^,^32^,^33^, and that information along with our MdtM model was used to guide identification of functionally related but not necessarily conserved residues in the latter^34^. This approach yielded an additional five residues (Q33, T120, M232, F261, and K342) as candidates for functioning in a substrate recognition/binding role in MdtM (Supplementary Fig. S3; Supplementary Table S3).

All candidate substrate-interacting residues were mutated individually to alter amino acid side chain charge and/or size, and the effects of each mutation on the efflux phenotype of *E. coli* that overexpressed each mutant transporter was tested to provide a guide for further studies that investigated: (i) binding of substrate to purified mutant protein; and (ii) transport activity of mutant protein in inverted vesicles. Additionally, to test our ‘aromatic clamp’ hypothesis, two double mutants (Y26A/Y123A and Y57A/F253A) were engineered and tested, and a Y26F/F253Y double mutant tested the effect of exchanging the position of these two aromatic residues.

### Effect of MdtM mutations on *E. coli* efflux phenotype

**Figure 3:**
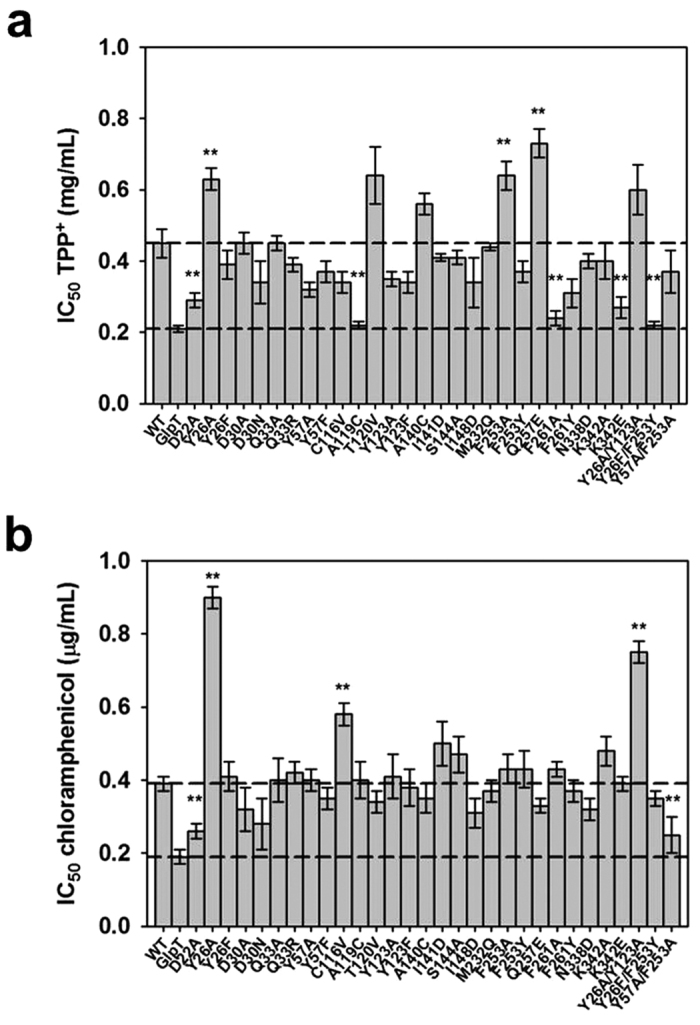
Antimicrobial efflux phenotypes of *E. coli* BW25113 cells that overexpressed wild type or mutant MdtM, or GlpT negative control, as determined by IC_50_ values. (**a**) IC_50_ values for TPP^+^ as antimicrobial substrate. (**b**) IC_50_ values for chloramphenicol as antimicrobial substrate. In (**a**,**b**) bars and error bars represent the mean ± s.d. of at least eight separate measurements. (**) denotes IC_50_ values that represent a statistically significant (P < 0.01) gain or loss of efflux function.

The results of the docking studies were used to inform design of a series of 30 MdtM mutants (27 single and 3 double mutants) and these mutant proteins were used in subsequent *in vivo* experiments to elucidate the role(s) of individual amino acid side chains in substrate recognition, binding, and transport. Initial studies focused on characterisation of the chloramphenicol and TPP^+^ efflux phenotypes of *E. coli* ∆*mdtM* single-deletion mutant cells that overproduced each mutant transporter from plasmidic DNA by determination of IC_50_ values for the substrates. Cells that overexpressed the MFS P_i_/glycerol 3-phosphate antiporter GlpT^35^ – a substrate-specific protein that does not possess ability to bind or transport chloramphenicol or TPP^+^-were used as a negative control; the mean (±s.d.) IC_50_ values for chloramphenicol and TPP^+^ in these negative control cells were 0.19 (±0.02) μg/mL and 0.21 (±0.01) mg/mL, respectively (Fig. 3). Cells that overexpressed plasmidic, wild type MdtM in the ∆*mdtM* background showed ~2-fold increase in resistance to both antimicrobials tested with IC_50_ values of 0.39 (±0.02) μg/mL for chloramphenicol and 0.45 (±0.04) mg/mL for TPP^+^. To exclude the effect that any significant differences in expression level of each protein could have on the IC_50_ values, Western blots of cell membranes were performed and these provided confirmation that expression levels of wild type and mutant MdtM protein and the GlpT negative control were similar (Supplementary Fig. S4).

Comparison of the IC_50_ values for TPP^+^ and chloramphenicol (Fig. 3a,b, respectively) revealed that mutation of MdtM residues suggested by the *in silico* docking studies to play a role in substrate binding tended to have a more pronounced effect on the *E. coli* TPP^+^ efflux phenotypes with the majority of the 30 MdtM mutants showing an increase in susceptibility towards the cationic antimicrobial (Fig. 3a). An analysis of the IC_50_ data was performed using an unpaired *t*-test to identify those mutations that resulted in a statistically significant decrease or increase in resistance to TPP^+^ compared to wild type MdtM. To avoid the effects of small differences in expression levels of MdtM influencing the analysis the alpha level was set to 0.01 and differences in IC_50_ values were considered to be significant if P < 0.01. Using this analysis, five of the MdtM mutants (D22A, A119C, F261A, K342E and the Y26F/F253Y double mutant) exhibited a significant loss of function compared to wild type protein, with IC_50_ values for TPP^+^ that did not differ significantly from that of the GlpT negative control (Fig. 3a). In addition to the loss of function efflux phenotypes, three of the MdtM mutations (Y26A, F253A, and Q257E) yielded a significant (P < 0.01) gain of function with respect to resistance against TPP^+^, with each showing ~1.5-fold increase in IC_50_ for the antimicrobial, and with the largest mean increase in resistance associated with the Q257E mutation.

In contrast to the generally deleterious effect that mutagenesis of MdtM had on the TPP^+^ IC_50_ values, the chloramphenicol efflux phenotypes appeared much more resilient towards mutagenesis and only two of the mutations tested (D22A and the Y57A/F253A double mutant) exhibited a significant (P < 0.01) loss of resistance to the antibiotic (Fig. 3b). Significant (P < 0.01) gain of function was observed for three mutants tested (Y26A, C116V, and the Y26A/Y123A double mutant) with a ~2.5-fold and ~2-fold increase in resistance to chloramphenicol conferred by the Y26A and Y26A/Y123A mutations, respectively. To ascertain whether the loss or gain of function observed from the efflux phenotype assays emanated from changes in substrate binding affinity and/or transport activity of the MdtM mutants, and to further test our ‘aromatic clamp’ hypothesis, we performed binding studies on purified protein in DDM detergent solution and transport measurements on inverted vesicles prepared from *E. coli* cells that overexpressed mutant transporter.

### Loss of function efflux phenotypes retained MdtM substrate-binding activity

Apparent binding dissociation constants (*K*_d_^app^) of purified wild type and mutant MdtM protein for TPP^+^ and chloramphenicol substrates were extracted from non-linear regression analysis of steady-state intrinsic tryptophan fluorescence quenching data (Supplementary Figs S5 and S6). The analysis revealed that loss of function observed in the efflux phenotype assays was not derived from a loss of MdtM substrate binding activity, as no single residue tested was essential for binding of either of the substrates to the protein (Tables 1 and 2). Substrate binding affinity of wild type protein for the substrates was in the nM range, with *K*_d_^app^ values for TPP^+^ and chloramphenicol of 400 (±145) nM (Table 1) and 22 (±4) nM (Table 2), respectively. The mutant proteins also possessed nM binding affinities and those mutants associated with gain of function efflux phenotype generally exhibited an increase in affinity for substrate compared to wild type protein. Intriguingly, the proposed TPP^+^ clamp (Y26/Y123), when substituted to alanine, appeared to drastically lower the *K*_d_^app^ for chloramphenicol to ~1 nM, whereas alanine substitution of the chloramphenicol clamp (Y57/F253) increased the *K*_d_^app^ of the neutral antibiotic about 17.5-fold (Table 2). Although the effect of the Y26A/Y123A double substitution on binding of TPP^+^ was not as dramatic and resulted in about a 2-fold decrease in *K*_d_^app^, the Y57A/F253A chloramphenicol clamp double mutation resulted in a significant decrease of the *K*_d_^app^ for the cationic substrate to ~56 nM (Table 1). However, in the latter case, the apparent tighter binding of TPP^+^ to the mutant did not translate into a gain of function efflux phenotype (Fig. 3a).

**Figure 4:**
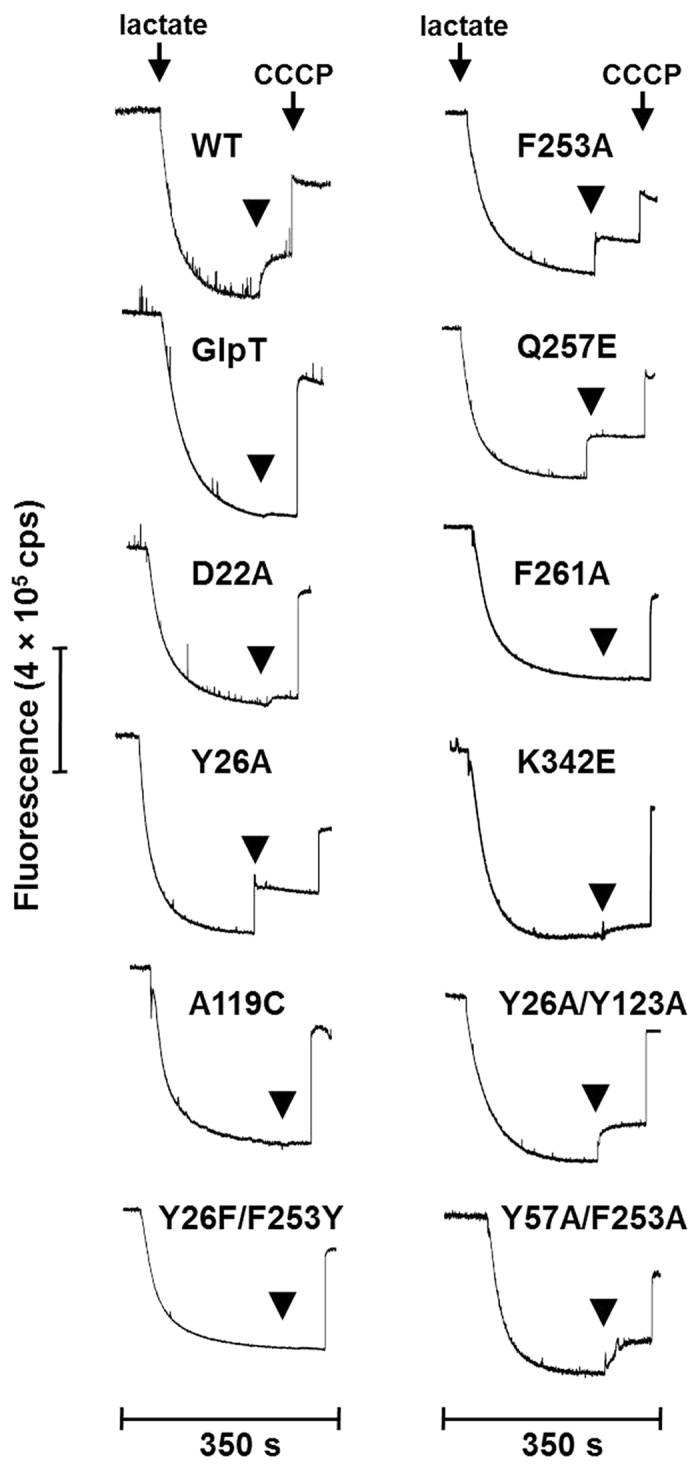
MdtM-dependent TPP^+^/H^+^ exchange in inverted vesicles. Transport measurements were performed by monitoring the fluorescence quench/dequench of acridine orange upon addition of TPP^+^ to inverted vesicles prepared from *E. coli* TO114 cells that overproduced recombinant wild type or mutant MdtM or, as a control, GlpT. Respiration-dependent generation of ΔpH (acid inside) was established by addition of lactate as indicated and once the fluorescence quench of acridine orange reached a steady state, TPP^+^ substrate was added. Addition of 100 μM CCCP at the time indicated completely dissipated ΔpH. The traces are representative of experiments performed in triplicate on at least two separate preparations of inverted vesicles.

The relationship between substrate binding affinity and loss of function efflux phenotype was less clear; while the A119C and Y57A/F253A mutations resulted in substantially decreased affinity for TPP^+^ and chloramphenicol, respectively, compared with wild type MdtM, the D22A, F261A and Y26F/F253Y mutants all bound TPP^+^ with affinity similar to that of the wild type protein (Tables 1 and 2). However, substitution of positively-charged K342 with acidic glutamate actually enhanced affinity of the transporter for TPP^+^ substrate (Table 1) even though transport capability of the mutant was severely compromised (Fig. 4). Retention of substrate binding activity by all the MdtM mutants tested indicated that loss of function efflux phenotype derived from transport dysfunction, probably due to inability of the protein to either undergo conformational change or facilitate proton transfer.

### Loss of efflux function stems from defects in the transport phase of the MdtM reaction cycle

**Figure 5:**
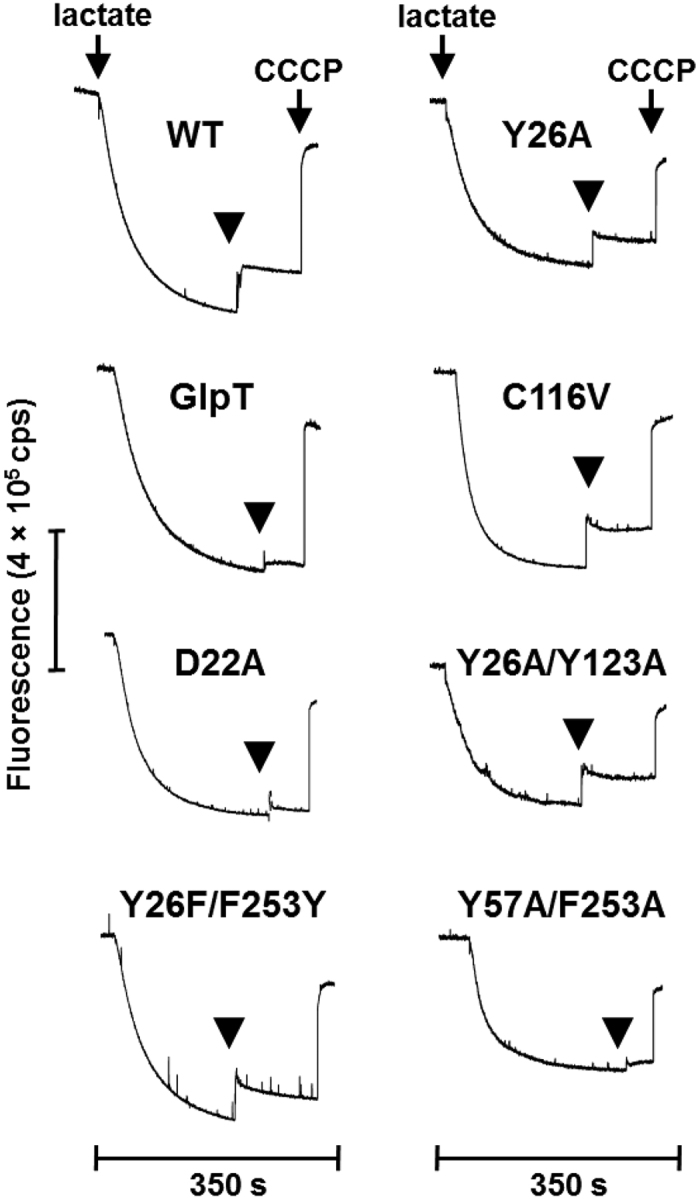
MdtM-dependent chloramphenicol/H^+^ exchange in inverted vesicles. Transport measurements were performed by monitoring the fluorescence quench/dequench of acridine orange upon addition of chloramphenicol to inverted vesicles prepared from *E. coli* TO114 cells that overproduced recombinant wild type or mutant MdtM or, as a control, GlpT. Respiration-dependent generation of ΔpH (acid inside) was established by addition of lactate as indicated and once the fluorescence quench of acridine orange reached a steady state, chloramphenicol substrate was added. Addition of 100 μM CCCP at the time indicated completely dissipated ΔpH. The traces are representative of experiments performed in triplicate on at least two separate preparations of inverted vesicles.

To examine the effects of mutagenesis on TPP^+^ and chloramphenicol efflux by MdtM we analysed transport activity of wild type and mutant transporter using a vesicle-based assay that reported proton-driven antiport activity as a dequench of acridine orange fluorescence. As shown in Figs 4 and 5, while vesicles prepared from cells that overexpressed wild type MdtM were clearly TPP^+^ and chloramphenicol transport-active (as revealed by ~20–30% dequench of the initial fluorescence signal upon addition of substrate), negative control vesicles that harboured GlpT produced negligible quenching of the fluorescence signal when TPP^+^ was used as substrate (Fig. 4). When chloramphenicol was added to the negative control, a small fluorescence dequench due to activity of chromosomally encoded transporters capable of efflux of the antibiotic was apparent (Fig. 5). In all the experiments, addition of the ionophore carbonyl cyanide 3-chlorophenylhydrazone (CCCP) to dissipate the proton electrochemical gradient caused an instantaneous dequenching of the fluorescence signal, thus providing evidence that the inverted vesicles had maintained integrity over the lifetime of the assay.

Although the D22A and K342E mutants appeared to retain some residual transport activity for TPP^+^, the A119C, F261A, and Y26F/F253Y mutations effectively abolished transport of the same substrate (Fig. 4), thus indicating the importance of these residues in MdtM for the translocation of cationic antimicrobials across the membrane. In contrast, the Y26A, F253A, and Q257E mutants exhibited enhanced TPP^+^ transport activity, with the Y26A and Q257E mutants in particular exhibiting a greater dequench of the initial fluorescence signal upon addition of the substrate than did wild type protein. Wild type transport activity for TPP^+^ was apparent for the Y26/Y123 and Y57/F253 double alanine substitutions (Fig. 4). Similarly, as shown in Fig. 5, while the MdtM Y26A, C116V, and Y26A/Y123A mutations associated with *E. coli* phenotypes that displayed gain of function with respect to chloramphenicol resistance retained ability to transport the antibiotic, the alanine substitutions of D22 and Y57/F253 resulted in almost total loss of chloramphenicol transport activity. Exchange of the positions of the tyrosine and phenylalanine residues in the Y26F/F253Y double mutant had negligible effect on chloramphenicol transport (Fig. 5). Taken together with the results of the efflux phenotype and substrate binding assays, these results (which are summarised in Supplementary Tables S4 and S5) clearly revealed that mutation of putative substrate-interacting residues in MdtM had a greater impact on the transport stage of the reaction cycle rather than the substrate-binding stage.

## Discussion

Recent crystal structures of *E. coli* multidrug efflux protein MdfA with substrate bound have placed previous biochemical studies of multidrug antiporters of the MFS in a structural context and have revealed the mode of substrate binding to the inward-facing conformation of the protein as well as suggesting a general transport mechanism^17^. However, it is unclear if the integrity of the binding site is retained or if different residues become involved in substrate binding as the transporter undergoes the conformational change necessary for transport. To investigate this, we explored the structural and functional features of the binding site(s) of MdtM, a close *E. coli* homologue of MdfA, in an occluded conformation that represented the protein during an intermediate stage of the transport cycle using a combination of *in silico* docking and site-directed mutagenesis studies. The observation that only three (Y26, Y57, and A119) of the 17 putative substrate-interacting residues identified by docking of TPP^+^ and chloramphenicol to MdtM were shown to interact with both substrates is consistent with the presence of distinct but partially overlapping binding sites to accommodate substrates of different structural and chemical properties within the transporter^15^,^36^.

With the exception of the significant increase in IC_50_ for both antimicrobials conferred by alanine substitution of Y26, and the apparent loss of efflux function associated with the D22A mutation in MdtM, other mutations that resulted in a gain or loss of TPP^+^ efflux function had no significant impact on the chloramphenicol efflux phenotype, and *vice versa* (Fig. 3). For example, although the A119C, F261A, K342E, and Y26F/F253Y mutations in MdtM resulted in loss of resistance to TPP^+^, the same mutations had no commensurate effect on resistance towards chloramphenicol. This implies that these residues are not crucial for transport activity of MdtM *per se* and that loss of TPP^+^ efflux function associated with the mutations originated from a defect in binding of the cationic substrate. However, because all the MdtM mutants retained TPP^+^ binding activity, abolition of efflux activity probably arose from the inability of TPP^+^-bound mutant to effect either conformational change or to release the cationic substrate. Interestingly, although F261 was not identified by our docking studies as a substrate-interacting residue we proposed a functional role for it based on comparison of our homology model of MdtM with the crystal structure of the MFS substrate-specific symporter FucP^37^. In FucP, a phenylalanine residue (F308) located in TM8 is important for transport function of that protein^38^, and F261 was located in a similar position on TM8 in our homology model of MdtM.

The differential effect of mutagenesis of the Q257 residue of MdtM on TPP^+^ and chloramphenicol efflux phenotypes may provide a clue as to why MdtM-catalysed transport of the latter appeared more resilient to the effects of mutation. Substitution of Q257 with glutamate resulted in an increase in both IC_50_ and binding affinity of cationic TPP^+^ compared with wild type protein but had no significant effect on IC_50_ for electrically neutral chloramphenicol. We propose that exchange of the amide side chain for an acidic one aids charge neutralisation of TPP^+^ to facilitate its export against the positive-outside membrane potential. Charge neutralisation of lipophilic cation substrates plays an important role in substrate recognition and binding in other multidrug binding proteins^39^,^40^ and it is likely to be a common mechanism employed by drug efflux representatives of the MFS. However, such a mechanism would not explain the differential effects of the F253A mutation; we speculate that in this case the gain of TPP^+^ efflux function was due to removal of a restrictive steric effect caused by the phenylalanine side chain, and that this promoted tighter binding. Similarly, increased binding affinity due to elimination of a sulfhydryl group within the substrate-binding pocket may explain the apparent differential impact of the C116V mutation on the chloramphenicol transport activity of MdtM.

The functional importance of acidic residues within the transmembrane spanning regions of MFS multidrug transporters is well established and the essential role of MdtM D22 in protection of *E. coli* cells against chloramphenicol and for efflux of cationic antimicrobial substrate by MdtM has been reported before^21^,^23^. Though the proton-titratable D34 residue of MdfA (which is homologous to MdtM D30) is critical for the substrate binding and transport activities of that protein^12^,^17^,^41^, the MdtM D30A or D30N mutations had no significant effect on either the TPP^+^ or chloramphenicol resistance phenotypes (Fig. 3). D34 is proposed to function as a critical component of the proton transfer system within MdfA, and its deprotonation upon substrate binding is the trigger for conformational change of the transporter^17^. The apparently non-essential nature of the equivalent residue in MdtM for efflux of either neutral or cationic substrate suggests that one or more other residues located within the transmembrane spanning region of MdtM possess capacity to compensate for the loss of D30 and implies that this conserved amino acid makes different contributions to the transport reaction in the different transporters.

A differential role for conserved residues within MdtM and MdfA was highlighted most strikingly by alanine substitution of a conserved tyrosine, Y26, in TM1 of MdtM. Alanine substitution of the equivalent residue (Y30) in MdfA abolished binding of chloramphenicol and it was proposed that Y30 might act as part of a proton-wire^17^. The gain of efflux function for both TPP^+^ and chloramphenicol conferred by the Y26A mutation clearly discounted a role for the tyrosine in proton transfer in MdtM. This contention was supported by the observation that removal of the hydroxyl group by replacement of Y26 with phenylalanine retained a wild type or even enhanced efflux phenotype. We suggest that removal of the bulky aromatic ring(s) in the Y26A and Y26A/Y123A mutants relieved a steric hindrance and that the enhanced efflux activity associated with the mutation(s) was a consequence of the increased affinity for substrate.

Docking studies revealed that Y26 and Y123 formed an ‘aromatic clamp’ around TPP^+^ in the MdtM substrate-binding site, and Y57 and F253 appeared to form a similar ‘clamp’ around chloramphenicol. Mutagenic exchange of the tyrosine and phenylalanine residues in the Y26F/F253Y mutant abrogated TPP^+^ efflux, whereas the same mutagenesis had no impact on chloramphenicol efflux phenotype. In contrast, while the Y57A/F253A double mutation severely compromised chloramphenicol transport, there was no substantial effect on TPP^+^ efflux activity. These observations suggest that the spatial arrangement of the tyrosine and phenylalanine residues is an important recognition factor for cationic and neutral antimicrobial substrates in MdtM. It is therefore tempting to speculate that ‘aromatic clamps’ within the substrate translocation pore of MFS multidrug efflux proteins may be an important structural/mechanistic feature of this class of transporter.

## Methods

### Homology modelling

The homology model of MdtM was generated using the ModPipe^42^ module of the ModWeb automated comparative modelling web-server (http://salilab.org/modbase)^43^. The amino acid sequence of MdtM from *E. coli* K-12 (UniProtKB P39386) was used as the target input and the ModWeb database of non-redundant chains of three-dimensional structures in the Protein Data Bank (PDB) used as structural templates. Initially, a number of models were built for each of the target sequence-template matches. The best-scoring models as assessed by ModPipe quality score (MPQS)^43^ and Discrete Optimized Protein Energy (DOPE) statistical potential^44^ were selected for further filtering and quality evaluation. Only those models with quality criteria values above specified thresholds or an *E*-value < 10^−4^ were included in the final model set; four of these were filtered as potential candidates for the final model and subjected to additional validation analyses using PROCHECK^45^ and WHAT IF^46^, and visualised by PyMol^47^. Based on these quality tests, the best-performing model was identified as one that used the crystal structure of the *E. coli* multidrug transporter EmrD (PDB code 2GFP)^16^ as the template. This model was loop-optimised with MODELLER^48^, validated again using PROCHECK^45^, and then used for molecular docking studies.

### Molecular docking studies

Molecular docking calculations on the MdtM protein model were carried out using DockingServer (http://www.dockingserver.com)^49^. The MMFF94 force field^50^ was used for energy minimization of the cationic substrate tetraphenylphosphonium (TPP^+^) and neutral substrate chloramphenicol (2,2-dichloro-N-[2-hydroxy-1-hydroxymethyl-2-(4-nitro-phenyl)-ethyl]-acetamide). Gasteiger partial charges were added to the ligand atoms. Essential hydrogen atoms, Kollman united atom type charges, and solvation parameters were added with the aid of AutoDock tools^51^. Affinity (grid) maps of 30 × 30 × 30 Å grid points and 0.375 Å spacing were generated using the Autogrid program^51^. AutoDock parameter set- and distance-dependent dielectric functions were used in the calculation of the van der Waals and the electrostatic terms, respectively. Docking simulations were performed using the Lamarckian genetic algorithm (LGA) and the Solis & Wets local search method^52^. Initial position, orientation, and torsions of the ligand molecules were set randomly. Each docking experiment was derived from 100 different runs that were set to terminate after a maximum of 2500000 energy evaluations. The population size was set to 150. During the search, a translational step of 0.2 Å, and quaternion and torsion steps of 5 were applied. For each substrate investigated, the docking result with the lowest free energy of binding was selected as final.

### Bacterial strains, plasmids and site-directed mutagenesis

*E. coli* BW25113 {*rrnB3* Δ*lacZ4787 hsdR514* Δ(*araBAD*)567 Δ(*rhaBAD*)568 *rph-1*} and its Δ*mdtM* single-deletion mutant were obtained from the Keio collection (National BioResource Project, Japan)^53^. The Δ*mdtM* deletion mutant was used as the background strain for determining IC_50_ values of cells expressing wild type and mutant MdtM, and GlpT from pBAD/*Myc*-His A vector (Life Technologies). For production of inverted vesicles used in transport assays, *E. coli* TO114 (a strain deficient in the NhaA, NhaB and ChaA antiporters) complemented with plasmidic DNA encoding mutant MdtM or GlpT was used. Overproduction of wild type and mutant MdtM, and GlpT for purification and subsequent use in substrate-binding assays was performed in *E. coli* LMG194 (KS272 Δ*ara714 leu*::Tn*10*) transformed with the appropriate plasmid^21^. MdtM mutants were produced using the QuikChange Lightning site-directed mutagenesis kit (Agilent Technologies) with wild type *mdtM*-containing pBAD/*Myc*-His A vector as the template. The fidelity of each mutant construct was verified by DNA sequence analysis.

### Determination of IC_50_ values

The IC_50_ values of chloramphenicol and TPP^+^ were determined using a plate-based, microtitre assay. An overnight culture of *E. coli* BW25113 Δ*mdtM* deletion mutant harbouring the plasmidic *mdtM* was used to inoculate 100 mL of LB liquid medium containing kanamycin (30 μg/mL) and carbenicillin (100 μg/mL), then incubated at 37 °C for 2 hours prior to decreasing the temperature to 30 °C. Overexpression of plasmidic MdtM was induced at OD_600_ of 0.7 by addition of 0.002% (w/v) L-arabinose and incubation was continued until OD_600_ of 1.0 was achieved. 10^4^ cfu of this culture were used to inoculate the test wells of a pre-prepared 96-well, flat bottom plate. Plates were prepared by adding 200 μl of LB broth containing the appropriate antibiotics and 0.002% (w/v) L-arabinose to each of the wells. 100 μl of chloramphenicol or TPP^+^ solution (at initial concentrations of 2.7 μg/mL and 4 mg/mL, respectively) was then added to the first column of the plate. After mixing by gentle aspiration, 100 μl of column one was withdrawn and added to column two. This serial two-fold dilution procedure was repeated for the remaining test columns. The last three columns of each plate were used as blanks. The plates were sealed with gas permeable covers and incubated overnight at 37 °C prior to reading the OD_600_ of each well with a plate reader. IC_50_s were calculated as described previously^22^.

### Western blots

Inner membranes of *E. coli* BW25113 ∆*mdtM* deletion mutant that overexpressed recombinant GlpT, and wild type and mutant MdtM were solubilised by addition of 2.0% (w/v) β-D-dodecylmaltopyranoside (DDM), incubated for 1 h at 4 °C then centrifuged at 20,000 × g for 20 min to pellet unsolubilised material. Supernatant containing 80 μg of solubilised membrane protein was loaded onto an SDS-PAGE gel of 12% acrylamide and electrophoresis performed at 180 V for 1 h prior to transfer of resolved protein to nitrocellulose membrane. Recombinant, histidine-tagged protein was detected using HisProbe-HRP and SuperSignal West Pico Chemiluminescent Substrate (Pierce Thermo Scientific) according to the manufacturer’s instructions and visualised with photographic film.

### Protein purification

Wild type and mutant MdtM, and GlpT was purified using a protocol described previously^54^.

### Substrate binding assays

Substrate-binding affinity of purified MdtM for chloramphenicol or TPP^+^ was determined using intrinsic tryptophan fluorescence quenching as described previously^22^ except that the buffer system consisted of 50 mM Tris-HCl, pH 8.0, 100 mM NaCl, 5% (v/v) glycerol and 0.1% (w/v) DDM. Any inner filter effect was corrected for as described in^55^. Data were fitted using non-linear regression analysis with SigmaPlot 10 (Systat Software) to allow determination of apparent dissociation constants.

### Vesicular transport assays

Assays of chloramphenicol/H^+^ and TPP^+^/H^+^ antiport were conducted by measuring the fluorescence quenching/dequenching of the pH-sensitive indicator acridine orange upon addition of substrate to energized inverted membrane vesicles generated from antiporter-deficient *E. coli* TO114 cells that overproduced recombinant MdtM or negative control GlpT as described before^24^.

Cells were grown and inverted vesicles were generated using protocols described before^22^ and the total membrane protein concentration of the vesicles was determined using the bicinchoninic acid assay (Thermo Scientific Pierce). Transport measurements were performed at 25 °C using a Fluoromax-4 fluorometer (Horiba). The assay mixture was excited at 492 nm, and the fluorescence emission recorded at 525 nm with excitation and emission slits set to 1.5 nm and 2.5 nm, respectively. Inverted membrane vesicles were added to reaction buffer (10 mM BisTris propane, pH 7.2, 5 mM MgSO_4_ and 1 μM acridine orange) in a quartz cuvette to a final concentration of 0.5 mg/mL membrane protein. Respiration-dependent generation of ΔpH (acid inside) was initiated by addition of 2 mM Tris-D-L-lactate and, once a stable ΔpH was established, chloramphenicol or TPP^+^ was added to a final concentration of 100 μM to assess transport activity. The fluorescence dequenching upon addition of substrate was monitored for 60 s prior to the addition of 100 μM carbonyl cyanide 3-chlorophenylhydrazone (CCCP) to completely dissipate ΔpH and abolish transport.

## Additional Information

**How to cite this article**: Alegre, K. O. *et al.* Insight into determinants of substrate binding and transport in a multidrug efflux protein. *Sci. Rep.* **6**, 22833; doi: 10.1038/srep22833 (2016).

## Supplementary Material

Supplementary Information

## Figures and Tables

**Table 1:** Binding affinity of wild type and mutant MdtM for TPP^+^.

MdtM protein	Apparent *K*_d_ (nM)	R-squared
WT	400 ± 145	0.96
D22A	449 ± 54	0.99
Y26A	147 ± 24	0.99
A119C	1083 ± 73	0.98
F253A	222 ± 40	0.97
Q257E	257 ± 76	0.98
F261A	415 ± 56	0.99
K342E	147 ± 11	0.96
Y26A/Y123A	206 ± 46	0.96
Y26F/F253Y	498 ± 52	0.98
Y57A/F253A	56 ± 10	0.98

*K*_d_^app^ values represent the mean ± s.d. of three measurements. R-squared values indicate the fit of a non-linear regression model to the substrate binding data presented in Supplementary Fig. S5.

**Table 2:** Binding affinity of wild type and mutant MdtM for chloramphenicol.

MdtM protein	Apparent *K*_d_ (nM)	R-squared
WT	22 ± 4	0.97
D22A	20 ± 4	0.96
Y26A	4 ± 1	0.96
C116V	4 ± 2	0.97
Y26A/Y123A	1 ± 0.5	0.97
Y26F/F253Y	18 ± 6	0.91
Y57A/F253A	387 ± 115	0.96

*K*_d_^app^ values represent the mean ± s.d. of three measurements. R-squared values indicate the fit of a non-linear regression model to the substrate binding data presented in Supplementary Fig. S6.

## References

[b1] Li X. Z. & Nikaido H. Efflux-mediated drug resistance in bacteria. Drugs. 64, 159–204 (2004). 14717618 10.2165/00003495-200464020-00004

[b2] Saier M. H. Jr. *et al.* The major facilitator superfamily. J. Mol. Microbiol. Biotechnol. 1, 257–279 (1999). 10943556

[b3] Law C. J., Maloney P. C. & Wang D. N. Ins and outs of major facilitator superfamily antiporters. Annu. Rev. Microbiol. 62, 289–305 (2008). 18537473 10.1146/annurev.micro.61.080706.093329 PMC2612782

[b4] Law C. J., Yang Q., Soudant C., Maloney P. C. & Wang D. N. Kinetic evidence is consistent with the rocker-switch mechanism of membrane transport by GlpT. Biochemistry. 46, 12190–12197 (2007). 17915951 10.1021/bi701383g PMC2435215

[b5] Pao S. S., Paulsen I. T. & Saier M. H. Jr. Major facilitator superfamily. Microbiol. Mol. Biol. Rev. 62, 1–34 (1998). 9529885 10.1128/mmbr.62.1.1-34.1998 PMC98904

[b6] Van Bambeke F., Balzi E. & Tulkens P. M. Antibiotic efflux pumps. Biochem. Pharmacol. 60, 457–470 (2000). 10874120 10.1016/s0006-2952(00)00291-4

[b7] Lewinson O., Adler J., Sigal N. & Bibi E. Promiscuity in multidrug recognition and transport: the bacterial MFS Mdr transporters. Mol. Microbiol. 61, 277–284 (2006). 16856936 10.1111/j.1365-2958.2006.05254.x

[b8] Neyfakh A. A. Mystery of multidrug transporters: the answer can be simple. Mol. Microbiol. 44, 1123–1130 (2002). 12068801 10.1046/j.1365-2958.2002.02965.x

[b9] Adler J. & Bibi E. Determinants of substrate recognition by the *Escherichia coli* multidrug transporter MdfA identified on both sides of the membrane. J. Biol. Chem. 279, 8957–8965 (2004). 14688269 10.1074/jbc.M313422200

[b10] Adler J. & Bibi E. Promiscuity in the geometry of electrostatic interactions between the *Escherichia coli* multidrug resistance transporter MdfA and cationic substrates. J. Biol. Chem. 280, 2721–2729 (2005). 15557318 10.1074/jbc.M412332200

[b11] Adler J., Lewinson O. & Bibi E. Role of a conserved membrane-embedded acidic residue in the multidrug transporter MdfA. Biochemistry. 43, 518–525 (2004). 14717607 10.1021/bi035485t

[b12] Edgar R. & Bibi E. A single membrane-embedded negative charge is critical for recognizing positively charged drugs by the *Escherichia coli* multidrug resistance protein MdfA. EMBO J. 18, 822–832 (1999). 10022825 10.1093/emboj/18.4.822 PMC1171175

[b13] Sigal N., Molshanski-Mor S. & Bibi E. No single irreplaceable acidic residues in the *Escherichia coli* secondary multidrug transporter MdfA. J. Bacteriol. 188, 5635–5639 (2006). 16855255 10.1128/JB.00422-06 PMC1540044

[b14] Zheleznova E. E. *et al.* A structure-based mechanism for drug binding by multidrug transporters. Trends Biochem. Sci. 25, 39–43 (2000). 10664577 10.1016/s0968-0004(99)01514-5

[b15] Lewinson O. & Bibi E. Evidence for simultaneous binding of dissimilar substrates by the *Escherichia coli* multidrug transporter MdfA. Biochemistry. 40, 12612–12618 (2001). 11601985 10.1021/bi011040y

[b16] Yin Y., He X., Szewczyk P., Nguyen T. & Chang G. Structure of the multidrug transporter EmrD from *Escherichia coli*. Science. 312, 741–744 (2006). 16675700 10.1126/science.1125629 PMC3152482

[b17] Heng J. *et al.* Substrate-bound structure of the E. coli multidrug resistance transporter MdfA. Cell Res. 25, 1060–1073 (2015). 26238402 10.1038/cr.2015.94 PMC4559816

[b18] Edgar R. & Bibi E. MdfA, an *Escherichia coli* multidrug resistance protein with an extraordinarily broad spectrum of drug recognition. J. Bacteriol. 179, 2274–2280 (1997). 9079913 10.1128/jb.179.7.2274-2280.1997 PMC178964

[b19] Nishino K. & Yamaguchi A. Analysis of a complete library of putative drug transporter genes in Escherichia coli. J. Bacteriol. 183, 5803–5812 (2001). 11566977 10.1128/JB.183.20.5803-5812.2001 PMC99656

[b20] Soo V. W., Hanson-Manful P. & Patrick W. M. Artificial gene amplification reveals an abundance of promiscuous resistance determinants in *Escherichia coli*. Proc. Natl. Acad. Sci. USA 108, 1484–1489 (2011). 21173244 10.1073/pnas.1012108108 PMC3029738

[b21] Holdsworth S. R. & Law C. J. Functional and biochemical characterisation of the *Escherichia coli* major facilitator superfamily multidrug transporter MdtM. Biochimie. 94, 1334–1346 (2012). 22426385 10.1016/j.biochi.2012.03.001

[b22] Holdsworth S. R. & Law C. J. The major facilitator superfamily transporter MdtM contributes to the intrinsic resistance of *Escherichia coli* to quaternary ammonium compounds. J. Antimicrob. Chemother. 68, 831–839 (2013). 23221628 10.1093/jac/dks491

[b23] Holdsworth S. R. & Law C. J. Multidrug resistance protein MdtM adds to the repertoire of antiporters involved in alkaline pH homeostasis in *Escherichia coli*. BMC Microbiol. 13, 113 (2013). 23701827 10.1186/1471-2180-13-113 PMC3668916

[b24] Paul S. *et al.* A single-component multidrug transporter of the major facilitator superfamily is part of a network that protects *Escherichia coli* from bile salt stress. Mol. Micro. 92, 872–884 (2014). 10.1111/mmi.12597 PMC4235344 24684269

[b25] Sigal N. *et al.* 3D model of the *Escherichia coli* multidrug transporter MdfA reveals an essential membrane-embedded positive charge. Biochemistry. 44, 14870–14880 (2005). 16274234 10.1021/bi051574p

[b26] Forrest L. R., Kramer R. & Ziegler C. The structural basis of secondary active transport mechanisms. Biochim. Biophys. Acta. 1807, 167–188 (2011). 21029721 10.1016/j.bbabio.2010.10.014

[b27] Law C. J., Enkavi G., Wang D. N. & Tajkhorshid E. Structural basis of substrate selectivity in the glycerol-3-phosphate: phosphate antiporter GlpT. Biophys. J. 97, 1346–1353 (2009). 19720022 10.1016/j.bpj.2009.06.026 PMC2749764

[b28] Law C. J. *et al.* Salt-bridge dynamics control substrate-induced conformational change in the membrane transporter GlpT. J. Mol. Biol. 378, 828–839 (2008). 18395745 10.1016/j.jmb.2008.03.029 PMC2426824

[b29] Fluman N., Cohen-Karni D., Weiss T. & Bibi E. A promiscuous conformational switch in the secondary multidrug transporter MdfA. J. Biol. Chem. 284, 32296–32304 (2009). 19808670 10.1074/jbc.M109.050658 PMC2781643

[b30] Madej M. G., Sun L., Yan N. & Kaback H. R. Functional architecture of MFS D-glucose transporters. Proc. Natl. Acad. Sci. USA 111, E719–727 (2014). 24550316 10.1073/pnas.1400336111 PMC3932877

[b31] Jeon J., Yang J. S. & Kim S. Integration of evolutionary features for the identification of functionally important residues in major facilitator superfamily transporters. PLoS Comput. Biol. 5, e1000522 (2009). 19798434 10.1371/journal.pcbi.1000522 PMC2739438

[b32] Baker J., Wright S. H. & Tama F. Simulations of substrate transport in the multidrug transporter EmrD. Proteins. 80, 1620–1632 (2012). 22434745 10.1002/prot.24056 PMC3349012

[b33] Guan L. & Kaback H. R. Lessons from lactose permease. Annu. Rev. Biophys. Biomol. Struct. 35, 67–91 (2006). 16689628 10.1146/annurev.biophys.35.040405.102005 PMC2802108

[b34] Madej M. G., Dang S., Yan N. & Kaback H. R. Evolutionary mix-and-match with MFS transporters. Proc. Natl. Acad. Sci. USA 110, 5870–5874 (2013). 23530251 10.1073/pnas.1303538110 PMC3625355

[b35] Huang Y., Lemieux M. J., Song J., Auer M. & Wang D. N. Structure and mechanism of the glycerol-3-phosphate transporter from *Escherichia coli*. Science. 301, 616–620 (2003). 12893936 10.1126/science.1087619

[b36] Putman M., Koole L. A., van Veen H. W. & Konings W. N. The secondary multidrug transporter LmrP contains multiple drug interaction sites. Biochemistry. 38, 13900–13905 (1999). 10529235 10.1021/bi991262k

[b37] Dang S. *et al.* Structure of a fucose transporter in an outward-open conformation. Nature. 467, 734–738 (2010). 20877283 10.1038/nature09406

[b38] Madej M. G. & Kaback H. R. Evolutionary mix-and-match with MFS transporters II. Proc. Natl. Acad. Sci. USA 110, E4831–4838 (2013). 24259711 10.1073/pnas.1319754110 PMC3864288

[b39] Peters K. M. *et al.* QacR-cation recognition is mediated by a redundancy of residues capable of charge neutralization. Biochemistry. 47, 8122–8129 (2008). 18616285 10.1021/bi8008246 PMC2646753

[b40] Vazquez-Laslop N., Markham P. N. & Neyfakh A. A. Mechanism of ligand recognition by BmrR, the multidrug-responding transcriptional regulator: mutational analysis of the ligand-binding site. Biochemistry. 38, 16925–16931 (1999). 10606527 10.1021/bi991988g

[b41] Fluman N., Ryan C. M., Whitelegge J. P. & Bibi E. Dissection of mechanistic principles of a secondary multidrug efflux protein. Mol. Cell. 47, 777–787 (2012). 22841484 10.1016/j.molcel.2012.06.018 PMC5524571

[b42] Eswar N. *et al.* Tools for comparative protein structure modeling and analysis. Nuc. Acid. Res. 31, 3375–3380 (2003). 10.1093/nar/gkg543 PMC168950 12824331

[b43] Pieper U. *et al.* ModBase, a database of annotated comparative protein structure models, and associated resources. Nuc. Acid. Res. 39, D465–474 (2010). 10.1093/nar/gkq1091 PMC3013688 21097780

[b44] Shen M. Y. & Sali A. Statistical potential for assessment and prediction of protein structures. Protein Sci. 15, 2507–2524 (2006). 17075131 10.1110/ps.062416606 PMC2242414

[b45] Laskowski R. A., Macarthur M. W., Moss D. S. & Thornton J. M. Procheck-a Program to Check the Stereochemical Quality of Protein Structures. J. Appl. Cryst. 26, 283–291 (1993).

[b46] Vriend G. What If-a Molecular Modeling and Drug Design Program. J. Mol. Graph. 8, 52 (1990). 2268628 10.1016/0263-7855(90)80070-v

[b47] DeLano W. L. & Lam J. W. PyMOL: A communications tool for computational models. Abstr. Pap. Am. Chem. S. 230, U1371–U1372 (2005).

[b48] Fiser A. & Sali A. Modeller: generation and refinement of homology-based protein structure models. Meth. Enzymol. 374, 461–491 (2003). 14696385 10.1016/S0076-6879(03)74020-8

[b49] Bikadi Z. & Hazai E. Application of the PM6 semi-empirical method to modeling proteins enhances docking accuracy of AutoDock. J. Cheminform. 1, 15 (2009). 20150996 10.1186/1758-2946-1-15 PMC2820493

[b50] Halgren T. A. Merck molecular force field.1. Basis, form, scope, parameterization, and performance of MMFF94. J. Comp. Chem. 17, 490–519 (1996).

[b51] Morris G. M. *et al.* Automated docking using a Lamarckian genetic algorithm and an empirical binding free energy function. J. Comp. Chem. 19, 1639–1662 (1998).

[b52] Solis F. J. & Wets R. J. B. Minimization by Random Search Techniques. Math. Op. Res. 6, 19–30 (1981).

[b53] Baba T. *et al.* Construction of *Escherichia coli* K-12 in-frame, single-gene knockout mutants: the Keio collection. Mol. Syst. Biol. 2, 2006 0008 (2006). 10.1038/msb4100050 PMC1681482 16738554

[b54] Alegre K. & Law C. Purification of a Multidrug Resistance Transporter for Crystallization Studies. Antibiotics. 4, 113–135 (2015). 10.3390/antibiotics4010113 PMC4790320 27025617

[b55] van de Weert M. Fluorescence quenching to study protein-ligand binding: common errors. J. Fluoresc. 20, 625–629 (2010). 19997966 10.1007/s10895-009-0572-x

