# Supplementary material for: Insight into determinants of substrate binding and transport in a multidrug efflux protein
Source: Sci Rep. 2016 Mar 10;6:22833. doi: 10.1038/srep22833 (PMC4785361; doi:10.1038/srep22833)
Supplement: Supplementary Information [file srep22833-s1.pdf]

Supplementary Information

for:

Insight into determinants of substrate binding and transport in a multidrug efflux protein

Kamela O. Alegre[1], Stephanie Paul[1], Paola Labarbuta[1] and Christopher J. Law[1,]*

[1]School of Biological Sciences, Medical Biology Centre, Queen's University Belfast, Belfast BT9 7BL, United Kingdom.

*Corresponding author: email: c.law@qub.ac.uk; Tel: 028 90972071; Fax: 028 90975877

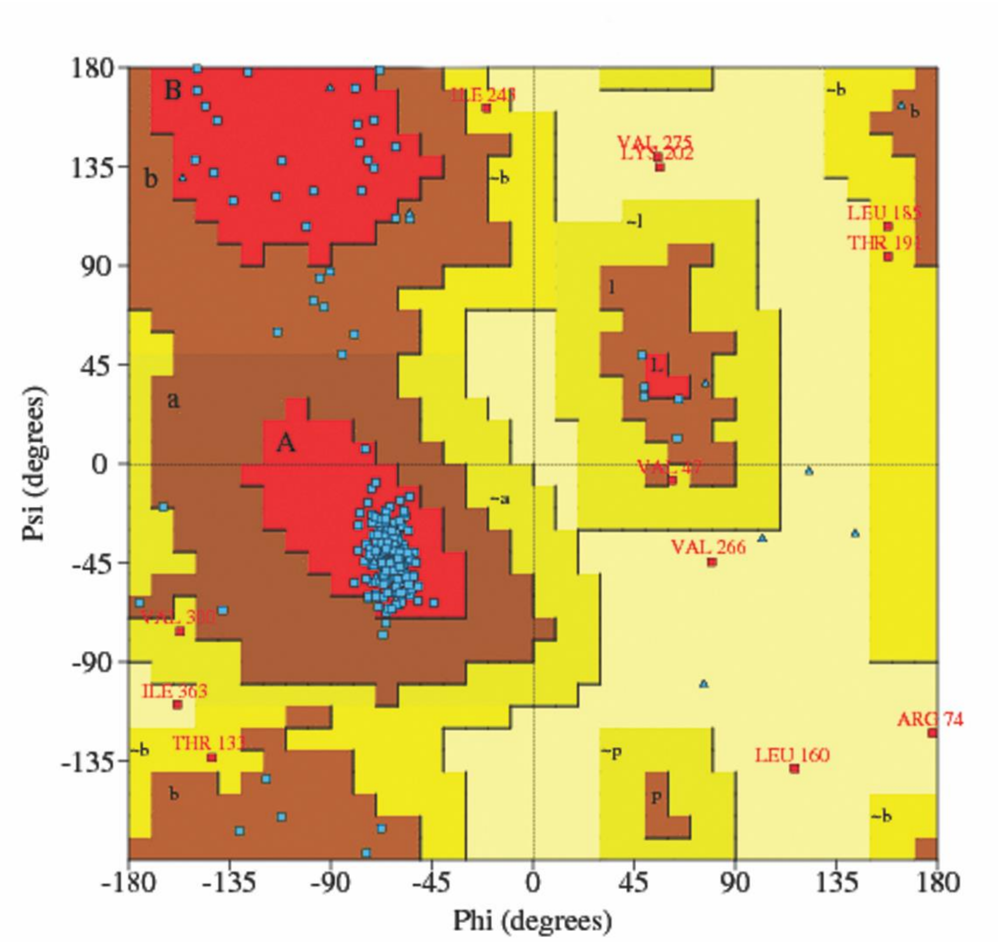

Figure S1 - Ramachandran plot of backbone dihedral angles for the homology model of MdtM in an occluded conformation. Individual amino acid residues are represented as turquoise squares or triangles or (for residues in disallowed regions) as red squares. A, B and L represent the most favoured regions of the plot; a, b, l and p represent additionally allowed regions; ~a, ~b, ~l and ~p represent generously allowed regions. Disallowed regions are shaded in light yellow.

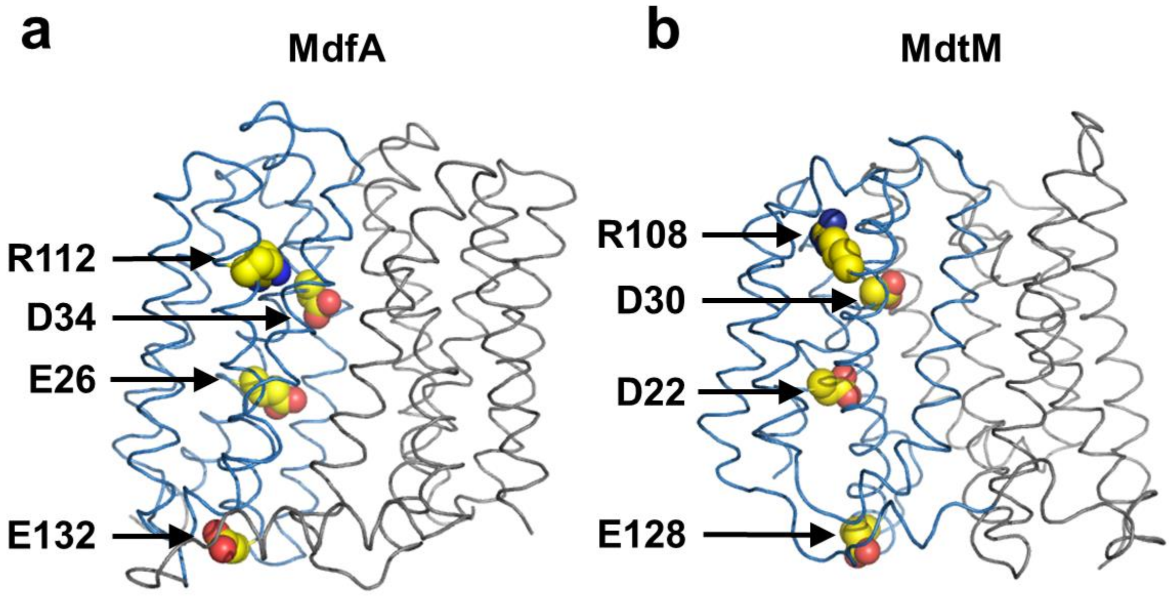

Figure S2 – Comparison of the locations of conserved, charged residues within the transmembrane spanning regions of the crystal structure of a substrate-bound, inward-facing conformation of MdfA (PDB: 4ZOW) and the occluded conformation homology model of MdtM. The view is parallel to the plane of the membrane with the periplasmic side of each protein at the top. The protein backbone is represented as ribbons and the highlighted residues as space-filling spheres. The N-terminal half of the protein is coloured sky blue and the C-terminal half in grey. Yellow spheres represent carbon atoms, blue spheres represent nitrogen atoms, and red spheres represent oxygen atoms. Figure was prepared using the PyMOL Molecular Graphics System (Schrödinger, LLC).

Table S1 – Interaction table detailing residue-ligand interactions between wild type MdtM and TPP^+.

Hydrogen bonds	Polar	Hydrophobic	π-π	Cation-π	Other
none	*none*	C24 [3.42] CYS116 (SG)	C24 [3.24] TYR26 (CB)	*none*	C20 [3.30] ASP22 (OD1)
		C20 [3.73] CYS116 (SG)	C20 [3.83] TYR26 (CB)		C12 [3.59] ASP22 (OD1,OD2)
		C9 [3.50] ALA119 (CB)	C18 [3.48] TYR123 (CB,CD2)		C6 [3.38] ASP22 (OD2)
		C2 [3.55] ALA119 (CB)	C10 [3.78] TYR123 (CD2)		C14 [3.16] ASP22 (OD2)
		C10 [3.74] ALA119 (CB)	C13 [3.85] TYR123 (CE2)		C22 [3.78] ASP22 (OD2)
		C4 [3.73] ALA119 (CB)			C21 [3.51] THR120 (CB, OG1)
		C20 [3.70] ALA119 (CB)			C17 [3.79] THR120 (CB)
		C12 [2.87] ALA119 (CB)			C6 [2.97] SER144 (CB, OG)
		C22 [3.78] ALA140 (CB)			C14 [3.19] SER144 (CB)
		C7 [3.57] ILE141 (CD1,CG2)			

Table S2 – Interaction table detailing residue-ligand interactions between wild type MdtM and chloramphenicol.

Hydrogen bonds	Polar	Hydrophobic	π-π	Cation-π	Other
N SER144<br>(9) (OG)<br>[3.24]	O ASP22<br>(7) (OD1, OD2)<br>[2.95]	C CYS116<br>(20) (SG)<br>[3.29]	C TYR26<br>(20) (CB)<br>[3.46]	H TYR26<br>(32) (CB)<br>[3.83]	O ASP22<br>(7) (CG)<br>[3.66]
O SER144<br>(7) (OG)<br>[3.14]	H ASP22<br>(32) (OD1, OD2)<br>[2.04]	C ILE148<br>(20) (CD1)<br>[3.22]	C TYR57<br>(20) (CZ)<br>[3.89]		H ASP22<br>(32) (CG)<br>[2.92]
	O TYR57<br>(7) (OH)<br>[3.27]		C PHE253<br>(20) (CE2)<br>[3.85]		O TYR26<br>(7) (CB)<br>[3.77]
	H SER144<br>(32) (OG)<br>[2.50]				C ASP30<br>(20) (CB, CG)<br>[3.80]
	O GLN257<br>(7) (NE2, OE1)<br>[2.87]				C TYR57<br>(20) (OH)<br>[3.53]
	H GLN257<br>(32) (NE2, OE1)<br>[2.02]				L ALA119<br>( ) (CB)<br>[3.82]
					O ALA119<br>(7) (CB)<br>[3.15]

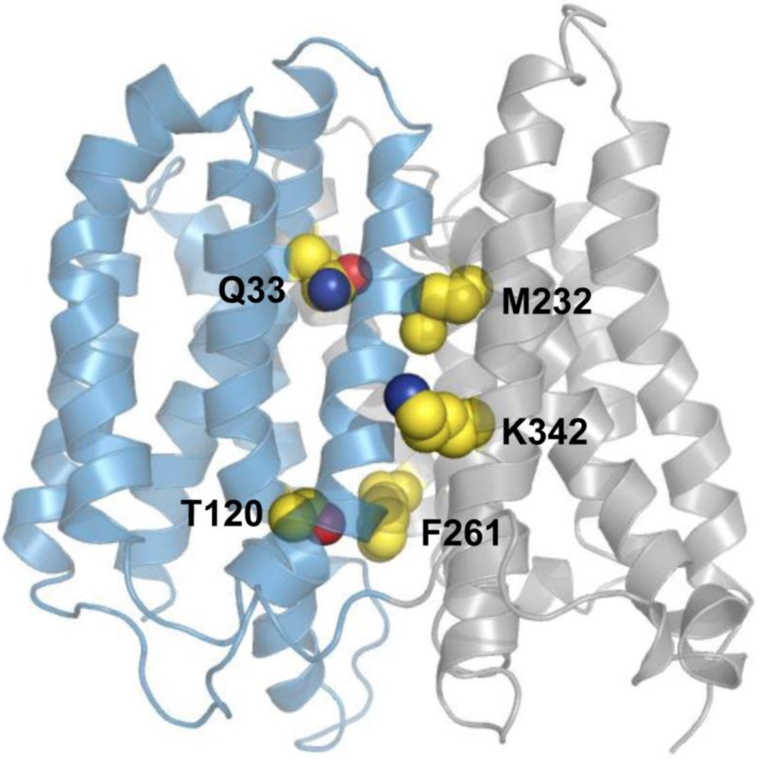

Figure S3 - Location of additional amino acid residues (represented as coloured spheres with carbon atoms coloured yellow, oxygen atoms red and nitrogen atoms blue) that could potentially function in substrate binding to MdtM. The N-terminal half of the protein is coloured sky blue and the C-terminal half in grey. The model is viewed parallel to the plane of the membrane with the periplasmic side of the protein at the top and the C-terminal cytoplasmic tail at the bottom right. Figure was produced using the PyMOL Molecular Graphics System (Schrödinger, LLC).

Table S3 - Amino acid residues suggested as candidates for involvement in substrate recognition and binding in MdtM.

Residue	Location on model	Reference
D22	TM1	1, 2, 4, 8, 9, and this work
Y26	TM1	9, and this work
D30	TM1	1, 9, and this work
Q33	TM1	6
Y57	TM2	5, 6, and this work
C116	TM4	This work
A119	TM4	This work
T120	TM4	This work
Y123	TM4	2, 3, 5, 7, and this work
A140	TM5	This work
I141	TM5	This work
S144	TM5	This work
I148	TM5	This work
M232	TM7	10
F253	TM8	This work
Q257	TM8	5, 6, and this work
F261	TM8	3, 11
K342	TM11	6

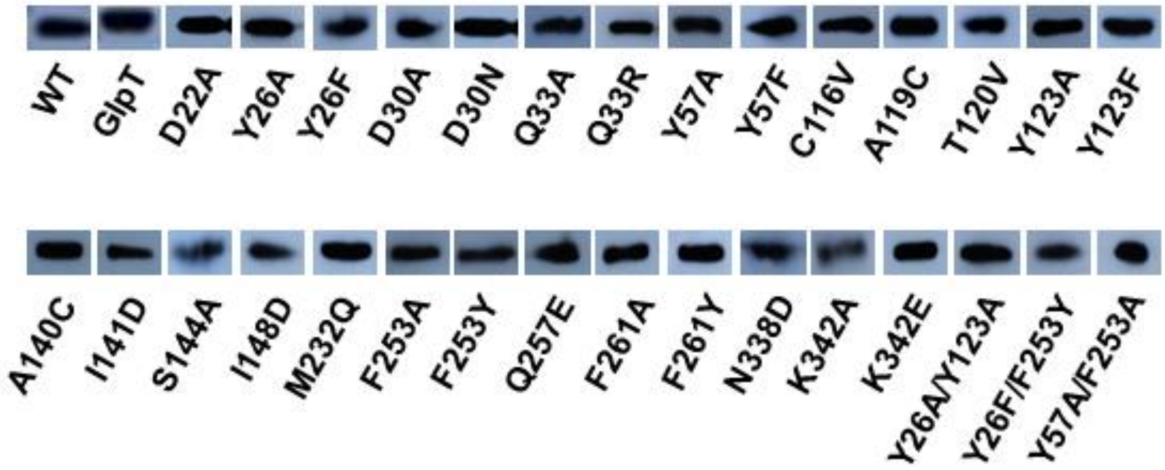

Figure S4 – Western blot analysis of expression levels of wild type MdtM, GlpT and the MdtM single and double mutants used in this study. Recombinant MdtM protein was visualized via detection of the C-terminal histidine tag by India His probe as described in the Methods. Each lane of the gels used for Western blots was loaded with 80 µg of DDM-detergent solubilised total membrane protein.

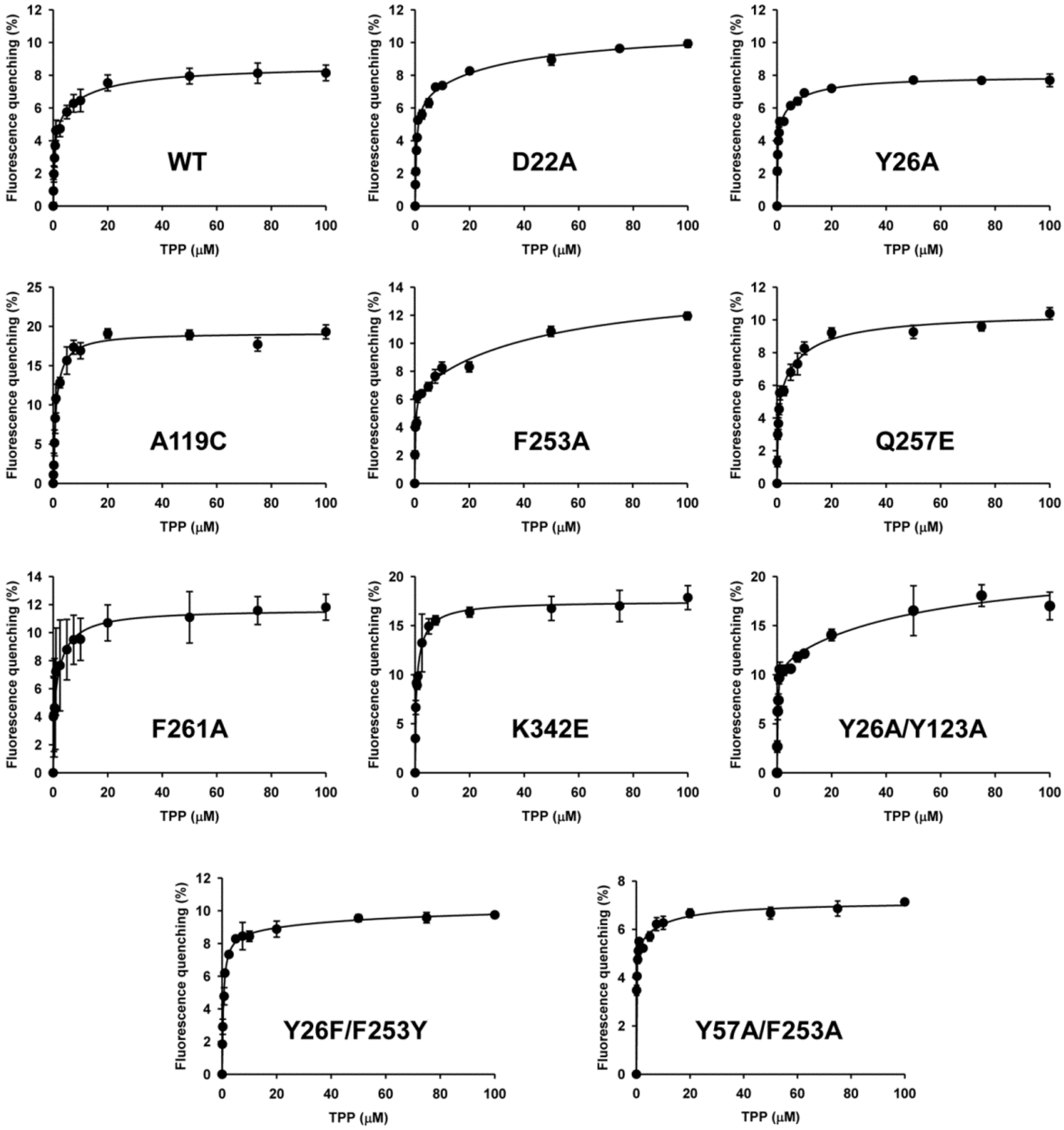

Figure S5 - TPP^+ binding by purified wild type and mutant MdtM in DDM detergent solution. Binding was measured by concentration-dependent intrinsic fluorescence quenching. Data points and error bars represent the mean ± s.d. of three measurements and data were fitted to a binding equation using non-linear regression (solid lines). The determined apparent dissociation constants (K_d^{app}) are reported in Table 1.

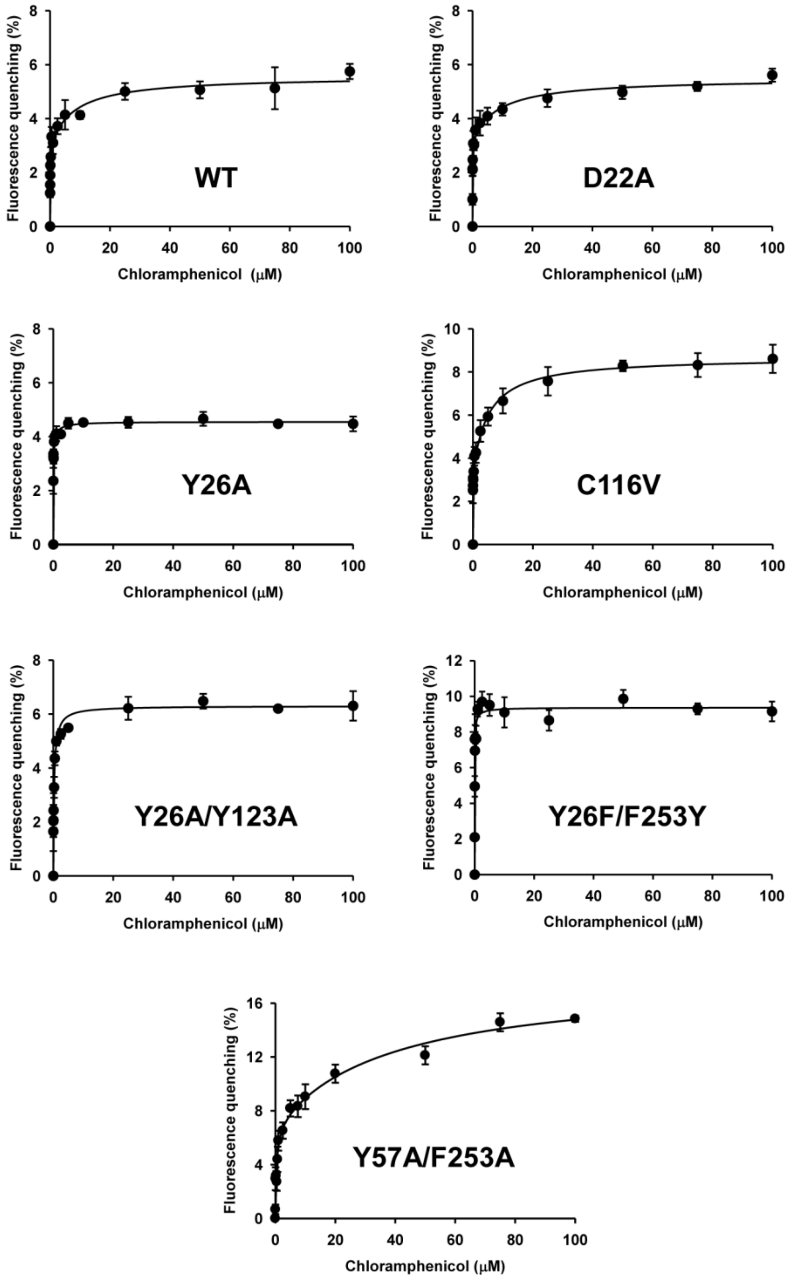

Figure S6 - Chloramphenicol binding by purified wild type and mutant MdtM in DDM detergent solution. Binding was measured by concentration-dependent intrinsic fluorescence quenching. Data points and error bars represent the mean ± s.d. of three measurements and data were fitted to a binding equation using non-linear regression (solid lines). The determined apparent dissociation constants (K_d^{app}) are reported in Table 2.

Table S4 – Summary of the effects of mutagenesis on the IC_{50} value, apparent substrate binding dissociation constant (K_d^{app}) and transport activity of MdtM for TPP^+ substrate. Downward-facing arrows (↓) indicate a decrease in value compared to that of wild type MdtM and upward-facing arrows (↑) indicate an increase in value. WT indicates the mutant exhibited no significant difference (P<0.01) in value compared to that of wild type MdtM.

**MdtM mutant**	**IC_{50}**	**K_d^{app}**	**Transport active?**
D22A	↓	WT	Residual
Y26A	↑	↓	Yes
A119C	↓	↑	No
F253A	↑	↓	Yes
Q257E	↑	↓	Yes
F261A	↓	WT	No
K342E	↓	↓	Residual
Y26A/Y123A	WT	↓	Yes
Y26F/F253Y	↓	WT	No
Y57A/F253A	WT	↓	Yes

Table S5 – Summary of the effects of mutagenesis on the IC_{50} value, apparent substrate binding dissociation constant (K_d^{app}) and transport activity of MdtM for chloramphenicol substrate. Downward-facing arrows (↓) indicate a decrease in value compared to that of wild type MdtM and upward-facing arrows (↑) indicate an increase in value. WT indicates the mutant exhibited no significant difference (P<0.01) in value compared to that of wild type MdtM.

MdtM mutant	IC_{50}	K_d^{app}	Transport Active?
D22A	↓	WT	Residual
Y26A	↑	↓	Yes
C116V	↑	↓	Yes
Y26A/Y123A	↑	↓	Yes
Y26F/F253Y	WT	WT	Yes
Y57A/F253A	↓	↑	Residual

References

1. Adler, J., Bibi, E. Role of a conserved membrane-embedded acidic residue in the multidrug transporter MdfA. *J. Biol. Chem*. **43**, 518-25 (2004).

2. Fluman, N., Cohen-Karni, D., Weiss, T., & Bibi, E. A promiscuous conformational switch in the secondary multidrug transporter MdfA. *J. Biol. Chem.* **284**, 32296-304 (2009).

3. Madej, M.G. & Kaback, H.R. Evolutionary mix-and-match with MFS transporters II. *Proc. Natl. Acad. Sci. U.S.A.* **110**, 4831-4838 (2013).

4. Sigal, N., et al. 3D model of the *Escherichia coli* multidrug transporter MdfA reveals an essential embedded positive charge. *Biochemistry*. **44**, 14870-80 (2005).

5. Baker, J., Wright, S.H. & Tama, F.L. Simulation of substrate transport in multidrug transporter EmrD. *Prot. Struc. Func. Bioinf.* **80**, 1620-32 (2012).

6. Jeon, J., Yang, J.S. & Kim, S. Integration of evolutionary features for the identification of functionally important members of the major facilitator superfamily transporters. *PLOS Comput. Biol.* **5**, e1000522 (2009).

7. Guan, L. & Kaback, H.R. Lessons from lactose permease. *Ann. Rev. Biophys. Biomol. Struct.* **30,** 67-91 (2006).

8. Madej, M.G., Sun, L., Yan, N. & Kaback, H.R. Functional architecture of MFS D-glucose transporters. *Proc. Natl. Acad. Sci. U.S.A.* **111,** 719-727 (2014).

9. Heng, J. *et al.* Substrate-bound structure of the *E. coli* multidrug resistance transporter MdfA. *Cell. Res*. **25**, 1060-1073 (2015).

10. Sun, L. *et al.* Crystal structure of a bacterial homologue of glucose transporters GLUT1-4. *Nature*. **490**, 361-366 (2012).

11. Dang, S. *et al*. Structure of a fucose transporter in an outward-open conformation. *Nature.* **467**, 734-738 (2010).
